# Supply chain concentration and corporate financialization

**DOI:** 10.3389/fpsyg.2022.934753

**Published:** 2023-01-03

**Authors:** Meifeng Zou, Xindong Zhang

**Affiliations:** ^1^School of Economics and Management, Taiyuan University of Science and Technology, Postdoctoral Station of Political Science, Shanxi University, Taiyuan, China; ^2^School of Economics and Management, Shanxi University, Taiyuan, China

**Keywords:** supply chain concentration, corporate financialization, market competitive power, business profitability, operating risk

## Abstract

We investigate whether firms that rely on major suppliers or customers, which is defined as supply chain concentration, tend to hold more financial assets than other firms due to supply chain pressure. We find that firms with a higher supply chain concentration have a higher financialization level. The firms' competitive power reduces their financialization level but cannot reverse the adverse impact of supply chain concentration. Furthermore, we explore the mechanism underlying the relationship between supply chain concentration and corporate financialization using the mediation effect method. We find that supply chain concentration impairs firms' main business profitability, leading firms to hold more financial assets. The main business profitability channels play partial mediating roles. Our findings reveal that overdependence on large suppliers/customers causes firms to escape reality and adopt virtual economics.

## Introduction

In China, there is a concerning phenomenon called the “escape from real to virtual economics,” which describes nonfinancial firms investing too many funds in financial assets (Peng et al., 2018). On the one hand, financial institutions allocate funds to the financial system instead of industrial sectors, leading to the circulation of financial capital in the financial system (Zhang and Zhang, 2016); on the other hand, nonfinancial firms reduce their investment in fixed assets but increase in financial products, such as stocks, bonds, and other financial derivatives; this phenomenon is called corporate financialization. If financial capital becomes the dominant form of capital, industrial capital may lose its dominant power in the value chain, which will lead to a decrease in real investment (Xie et al., 2014), restrain enterprises' R&D investment (Wang et al., 2017), and even affect economic growth (Zhou and Xie, 2018). Therefore, it is very important to explore which factors affect corporate financialization and how to prevent firms from being trapped in financialization.

A growing body of literature discusses corporate financialization, including its definition (Krippner, 2005; Stockhammer, 2005; Orhangazi, 2008), measurement (Demir, 2009; Shin and Zhao, 2013; Du et al., 2017), adverse impact (Orhangazi, 2008; Tori and Onaran, 2017), and beneficial impact (Aivazian et al., 2005; Denis and Sibilkov, 2009; Hsieh and Klenow, 2009; Kliman and Williams, 2014); the motivations for financialization (Smith and Stulz, 1985; Arrighi, 1996; Duchin et al., 2017); and how to address nonfinancial firms' tendency to financialize (Toporowski, 2000; Stockhammer, 2005; Orhangazi, 2008). However, literature on the factors affecting financialization is scarce; only Peng et al. (2017, 2018) study how economic policy uncertainty and tax reduction impact financialization and Du et al. (2019) find that executives with a financial background tend to hold more financial assets than those without such a background. This paper will explore whether and how a firm's supply chain concentration affects firm financialization. The supply chain concentration is defined as the sales or purchase ratio from a major supplier or customer, respectively; when the ratio is higher, it implies that the supply chain is more concentrated for a firm.

There are good reasons for focusing on the relationship between a firm's supply chain concentration and its financialization. Suppliers and customers are the most basic and important business relationships for the production and operation of a company (Dhaliwal et al., 2016). When the firm's supply chain concentration increases, the supplier or customer will gain stronger bargaining power (Chu, 2012; Patatoukas, 2012), which will have adverse impacts on the firm (Zhang et al., 2020). Suppliers may reduce trade credit, shorten trade credit terms, and even require advanced payments (Jorion and Zhang, 2009). Alternatively, when a supplier suffers a shock, the firm's normal operations will be disrupted (Cen et al., 2017). Supportive evidence suggests that if a firm relies on a large supplier, the firm will hold more cash (Zhang et al., 2020), reduce R&D expenditures (Ren and Zhang, 2019), etc. Customers are the main revenue source for firms (Gosman and Kohlbeck, 2009). The firm faces severe risk if major customers switch to other suppliers, enter the upstream industry, become financially distressed, or declare bankruptcy (Dhaliwal et al., 2016). A set of empirical studies shows that a firm that depends on large customers will suffer more negative effects, such as facing strict bank loan contracts (Campello and Gao, 2017), paying more equity capital (Dhaliwal et al., 2016), holding more cash (Itzkowitz, 2013), and lowering profitability (Irvine et al., 2013). The above adverse impact will impair firms' operations and, in turn, lead to decreased firm profitability. However, how the adverse influence of large suppliers and customers affects corporate financialization is unclear. We aim to explore the relationship between supply chain concentration and firm financialization and their underlying mechanisms.

First, we examine how supply chain concentration affects firm financialization. We use the ratio of the purchases and sales of the major suppliers and customers, respectively, as proxy variables for the supply chain concentration and measure firm financialization as the ratio of financial assets to total assets or financial returns to total income. We find that there is a significant positive link between supply chain concentration and financialization, which means that a firm with a higher supply chain concentration will hold more financial assets and gain more financial returns. This finding implies that when a firm faces a higher supply chain concentration, it may suffer pressure from both upstream (suppliers) and downstream enterprises (customers), reducing its profitability from production and operation activities. Therefore, these firms will begin to invest in financial assets instead of production assets.

Second, we consider the firm's competitive power and regress the model with competitive power. We find that there is a significant negative relationship between competitive power and firm financialization, which implies that if a firm can gain enough return from production and operation activities, it will reduce investments in financial products. However, the cross-term of supply chain concentration and competitive power still has a positive impact on financialization, which means that the firm suffers a more adverse impact from supply chain concentration than the firm's competitive power.

Third, we explore the underlying mechanism between supply chain concentration and firm financialization. We find that as a firm's supply chain concentration increases, the main profitability of the firm decreases. We verify that a firm's supply chain concentration impairs its profitability. Therefore, firms with higher supply chain concentrations will switch to holding more financial assets or products, that is, to financialization.

Finally, estimates of the relationship between supply chain concentration and firm financialization are subject to empirical biases. For instance, firms with a high level of financial assets tend to trade with major suppliers and customers due to their stable relationship, and other unobserved characteristics may cause a firm's supply chain concentration and financialization to increase. To alleviate concerns about estimation biases, we use the industrial supply chain concentration and a 1-year lag of the supply chain concentration as instrumental variables for the supply chain concentration. Our instrumental variable method estimations confirm that the baseline results are credible. A positive relation still exists between supply chain concentration and firm financialization. We also use other robustness tests to confirm our results, including alternatives to the independent and dependent variables and PSM procedures. All these tests show that our findings are reliable.

Our paper is related to several strands of the literature. First, we extend the literature on supplier-customer relationships. The prior literature pays more attention to the effect of customer concentration on firms' financial decisions, including the cost of capital (Cen et al., 2016), loan contract terms (Campello and Gao, 2017), relationship-specific investments (Kale and Shahrur, 2007), mergers and acquisitions (Ahern and Harford, 2014), and financial distress (Hertzel et al., 2008). However, they ignore how suppliers and customers together impact the firm's financial behavior. We explore whether and how supply chain concentration influences corporate financialization.

Second, our paper contributes to the recent hot topic on the determinants of corporate financialization. The existing literature concerning financialization pays more attention to the motivations for financialization (Krippner, 2005; Duchin et al., 2017), the impact of financialization (Denis and Sibilkov, 2009; Kliman and Williams, 2014; Tori and Onaran, 2017), and how to address nonfinancial firms' tendency to financialize (Toporowski, 2000; Stockhammer, 2005; Orhangazi, 2008). However, they overlook the effect of suppliers and customers, which are important business partners that also exert a significant impact on a firm's financialization. This paper explores the relationship between a firm's supply chain concentration and its financialization.

The remainder of this paper is organized as follows. Section Hypothesis development develops testable hypotheses. Section Data and variables describes the sample and variables. In Section Supply chain concentration and corporate financialization, we present the main empirical results. Section Mechanism analysis discusses the underlying mechanism driving our findings. Section Robustness tests presents additional robustness tests. In Section Further tests, we show further tests. Section Conclusion concludes the paper.

## Hypothesis development

This section formalizes the testable hypotheses on supply chain concentration and firm financialization. A firm with a major supplier and customer (i.e., supply chain concentration) may suffer some adverse effects. We divide the supply chain concentration into customer concentration and supplier concentration.

First, we focus on customer concentration. Having a concentrated customer base can bear negative implications for a firm. Major customers often require the firm to commit to relationship-specific investments (Banerjee et al., 2008). These investments cater to the need of big customers. But the firm conducting these investments faces a higher uncertainty of success and limited resales options of the output to alternative users. Accordingly, to spread risk, the firm holds more financial assets. Furthermore, the existing literature also argues that large customers often attain higher bargaining power over purchase prices and the timing of payments (Campello and Gao, 2017). They exert price pressure on their upstream firms, who in turn, are forced to hold financial investments due to customer risk. Firms that are dependent on major customers also face the risk of losing sales when these customers are in financial distress or transfer to other suppliers. As a result, these firms will hold more financial assets due to precautions (Itzkowitz, 2013). Taken together, the existing literature shows that corporate customer concentration can lead to more specific investments and operational risk, both of which can induce a higher level of financialization. Anticipating a higher level of financialization is associated with customer concentration.

Second, we focus on supplier concentration. A firm with a large supplier could be trapped in a passive position (Galbraith, 1952; Stigler, 1964; Snyder, 1996). Consequently, the supplier makes unreasonable requirements on the firm during their business negotiations due to the supplier's higher bargaining power. For instance, a firm may be forced to provide more commercial credit to big suppliers. The above behaviors will weaken the profitability of the firm which, in turn, has to hold more financial assets to gain profit. Furthermore, a business-to-business relationship has costs for the firm as well. The relationship has an implicit contact between them. A firm faces the risk of losing substantial future material if the supplier becomes financially distressed or declares bankruptcy, switches to a rival firm, or decides to enter a downstream industry, so the firm should maintain its financialization level to avoid the risk of suppliers making unexpected changes.

As a result, firms are more inclined to hold the financial investment to address the operational risks and cash flow risks introduced by the supply chain concentration. Scholars verify that low-performing companies will invest their limited funds in financial assets and use financial earnings to compensate for or even reverse the losses to their main businesses. Wang et al. (2017) also found that the decline in profitability caused by market competition is an important motivation for enterprises to make financial investments. From the perspective of the liquidity of financial assets, to prevent the cash flow risk caused by the over-concentration of customers and suppliers, enterprises will give priority to the allocation of funds to financial assets. Research by Peng et al. (2017) shows that when monetary policy tends to tighten, enterprises will increase the level of financial asset allocation and improve liquidity. Zhang and Zhang (2016) believe that financial assets are important tools used by enterprises to cope with capital shortages.

Based on the above analysis, we speculate that if the supply chain concentration of firms is high, enterprises are more inclined to use funds for financial investment than an industrial investment to prevent possible operational risks and cash flow risks in the future, exacerbating the trend of “from real to virtual.” Thus, we propose the following hypothesis: a positive correlation exists between the supply chain concentration and financialization for firms, i.e., the higher the supply chain concentration, the higher the financial asset holding (i.e., financialization) level of a firm.

## Data and variables

The suppliers/customers, financialization, and other accounting index datasets used in our analysis are assembled using the Chinese Stock Market and Accounting Research Database (CSMAR), which contains information regarding all suppliers and customers and financial data. We match the supplier/customer dataset with financialization data using the unique stock ID and year. Our sample period is from 2007 to 2021 because the number of observations before 2007 in CSMAR is insufficient. We exclude financial firms and firms without supplier/customer and financialization information. Our final sample consists of 4,855 firms with 43,692 firm-year observations. We winsorized on both tails of their distributions at 1%.

Our key variables used in this study are defined as follows. We use two financialization measures. One is the ratio of financial assets to total assets (FIN1); financial assets include monetary resources, trading financial assets, derivative financial assets, available-for-sale financial assets, held-to-maturity investment, and investment in real estate, following the prior literature (Crotty and Epstein, 1999; Krippner, 2005; Stockhammer, 2005; Orhangazi, 2008). The other is the ratio of investment earnings to total income (FIN2), where investment income equals the profit (loss) arising from fair value changes and income from investment. We use the supplier and customer concentrations to measure the supply chain concentration. The definition of supplier concentration inspired by customer concentration was proposed by Dhaliwal et al. (2016) and Campello and Gao (2017). Our first supplier concentration (SC1) is the purchase ratio from the largest supplier, and the second (SC2) is the purchasing ratio from the top five suppliers. The measurement of customer concentration is similar to that of supplier concentration; customer concentration is measured using CC1 (the sales ratio for the largest customer) and CC2 (the sales percentage from the top five customers). The other variables include return on assets (roa), leverage (lev), growth for sales (growth), etc. (see Table 1 for detailed descriptions of the variables).

**Table 1:** Variable definitions.

**Variable categories**	**Variable**	**Definitions**
Financialization	FIN1	The ratio of financial assets to total assets
	FIN2	The ratio of financial income to total income
Supply chain concentration	SC1	The proportion of purchases from the largest supplier
	SC2	The proportion of purchases from the top 5 suppliers
	CC1	The proportion of sales to the largest customer
	CC2	The proportion of sales to the top 5 customers
Control variables	lev	The book value of the total debt divided by the total assets
	zzl	The ratio of revenue to average total assets
	ldbl	The ratio of current assets to liquidity liabilities
	zbmjd	Sales revenue divided by the sum of the fixed assets and projects under construction
	roa	The book value of the operating profit divided by assets
	soe	Dummy variable equalling one if the firm is a state-owned enterprise and zero otherwise
	top1	The equity proportion of the largest shareholder
	size	The natural logarithm of the book value of assets
	cflow	The net cash flow from operating activities divided by the net assets
	age	The logarithm of the birth year of the firms

Table 2 reports the summary statistics of the supplier/customer concentration and firm characteristics in our sample. Firms that hold financial assets increase their total assets by 5.89% on average. Approximately 30% of the sample firms' sales and purchases are attributed to major customers and suppliers. For these firms, more than 10% of their purchases and sales are attributed to their largest supplier and customer, respectively, on average. This implies that there is a common phenomenon of supply chain concentration. It is necessary to explore the effect of supply chain concentration on firms' financial decisions.

**Table 2:** Descriptive statistics.

**Variable**	***N***	**Mean**	**SD**	**Min**	**p50**	**Max**
FIN1	43,596	5.890	8.980	0	3.030	99.79
SC2	43,596	22.89	23.80	0	18.82	93.34
SC1	43,596	6.740	11.43	0	0	56.38
CC2	43,596	25.58	24.36	0	19.79	98.28
CC1	43,596	8.170	13.09	0	2.770	68.07
lev	43,596	0.440	0.220	0	0.430	1
ldbl	43,596	2.460	2.620	0	1.620	16.17
zzl	43,596	38.67	140.6	0	5.670	1078
zbmjd	43,596	5.120	10.40	0	2.250	74.91
roa	43,596	0.040	0.080	−0.33	0.040	0.23
size	43,596	21.98	1.38	10.84	21.84	25.93
cflow	43,596	0.080	0.18	−0.60	0.080	0.69
top1	43,596	18.51	20.86	0	13.27	99
soe	43,596	0.200	0.400	0	0	1
age	43,596	7.600	0.190	0	7.600	7.610

This table presents the means, standard deviations, percentiles, and a number of observations of the variables used in the regression. The sample covers the 2007–2021 window. All continuous variables are winsorized within the 1st and 99th percentiles (see this table for the variable definitions).

## Supply chain concentration and corporate financialization

### The baseline regression

Suppliers and customers along the supply chain provide necessary inputs and income and are important for a firm's success. We examine whether a firm's supply chain concentration affects its financialization level by estimating an ordinary least squares (OLS) model using Equation (1):


(1) $$\begin{array}{l} {\text{Fin}}_{i,t}\;=\;\alpha \;+\;\beta_{1}SC_{i,t}\;+\;\beta_{2}Controls_{i,t}\;+\;\beta_{3}Ind\\ \;+\;\beta_{4}Year\;+\;\varepsilon_{i,t}, \end{array}$$

where *Fin*_*i, t*_ stands for one of the two financialization variables defined above for firm i in year t. The independent variable of *SC*_*i, t*_ is one of two supply chain concentration variables (i.e., SC2 and CC2) defined above. *Controls*_*i, t*_ represents a set of control variables that are commonly used in the financialization literature (see, e.g., Duchin et al., 2017). In all tests, we include year fixed effects to control for macroeconomic trends, province fixed effects to capture area differences across China, and industry fixed effects to capture differences across industries, following the approach in Akkemik and Özen (2013). The industry is classified according to the 3-digit CSRC industry standard. We cluster stand errors at the firm level.

Table 3 presents the results of the baseline regression (1). It provides clear evidence supporting our hypothesis that supply chain concentration measured by SC2 and CC2 positively affects a firm's financialization level. The coefficients on SC2 and CC2 are positive and highly significant when we control for firm, industry, year, and province fixed effects. For instance, the coefficients on SC2 in columns (1)–(3) are 0.0451, 0.058, and 0.0393, and the corresponding coefficients on CC2 are similar at 0.0373, 0.0505, and 0.0314. The results indicate that when a firm's purchases or sales are largely concentrated in one or several major suppliers or customers, the firm increases its financial assets.

**Table 3:** Financialization and supply chain concentration.

	**(1)**	**(2)**	**(3)**	**(4)**	**(5)**	**(6)**
	**FIN1**	**FIN1**	**FIN1**	**FIN1**	**FIN1**	**FIN1**
SC2	0.0451***	0.0580***	0.0393***			
	(22.1613)	(38.7154)	(19.0719)			
CC2				0.0373***	0.0505***	0.0314***
				(19.0121)	(28.9041)	(15.7138)
lev		−6.8530***	−4.8151***		−7.3000***	−4.8601***
		(−22.4005)	(−16.2890)		(−23.6943)	(−16.4177)
ldbl		−0.2119***	0.1547***		−0.2450***	0.1601***
		(−10.1703)	(7.3391)		(−11.6572)	(7.5858)
zzl		−0.0007**	0.0000		−0.0005*	0.0004
		(−2.4047)	(0.0166)		(−1.6604)	(1.3753)
zbmjd		−0.0047	−0.0123***		−0.0070*	−0.0101**
		(−1.1803)	(−2.8550)		(−1.7431)	(−2.3430)
roa		−4.2497***	−0.4112		−5.3320***	−0.5197
		(−8.2847)	(−0.6798)		(−10.3123)	(−0.8581)
size		0.4856***	−0.1691***		0.7607***	−0.1768***
		(11.2650)	(−4.5059)		(17.4424)	(−4.6861)
cflow		−0.0312	−0.7816***		0.0334	−0.7282***
		(−0.1666)	(−3.2884)		(0.1771)	(−3.0586)
top1		−0.0497***	−0.0324***		−0.0488***	−0.0326***
		(−23.7735)	(−9.8335)		(−23.1039)	(−9.8733)
soe		1.7789***	1.8081***		1.6926***	1.7435***
		(14.0875)	(14.0997)		(13.2959)	(13.5778)
age		0.0001	−2.1e+02***		0.0003	−2.1e+02***
		(0.0021)	(−12.6188)		(0.0081)	(−12.6353)
Firm FE	N	Y	N	N	Y	N
Year FE	Y	Y	Y	Y	Y	Y
Ind FE	Y	N	Y	Y	N	Y
Prov FE	Y	Y	Y	Y	Y	Y
Adj. *R*^2^	0.1093	−0.0502	0.1341	0.1067	−0.0678	0.1317
*F*	491.1214***	277.8565***	159.5179***	361.4607***	209.4025***	148.5605***
*N*	43,463	43,596	43,463	43,463	43,596	43,463

This table reports the relation between financialization and supply chain concentration. The independent variable is supply chain concentration, measured by SC2 and CC2. The dependent variable is financialization, measured by fin1, which is defined as the ratio of financial income to total income. All regressions use industry and year-fixed effects. The industry is classified by the 3-digit CSRC industry standard. Continuous variables are winsorized at their 1st and 99th percentiles. Standard errors are corrected for heteroskedasticity and clustering at the firm level (t-statistics are shown in parentheses), *p < 0.1, **p < 0.05, and ***p < 0.01. See Table 2 for the variable definitions.

t-statistics in parentheses, *p < 0.1, **p < 0.05, ***p < 0.01.

Consistent with prior literature (Peng et al., 2018), Table 3 shows that the debt to assets ratio (*lev*), tangible asset ratio (*zbmjd*), return on assets (roa), and the equity proportion of the largest shareholder (*top1*) tend to have a negative impact on a firm's financialization level. Thus, firms with a higher financial leverage ratio, higher tangible asset ratio, higher return on asset ratio, etc., tend to hold fewer financial assets. However, the key point is that these determinants of corporate financialization documented in the previous literature cannot subsume the effect of the supply chain concentration variable on firm financialization.

### Supply chain concentration, market competitive power, and financialization

The above empirical evidence suggests a positive relationship between supply chain concentration and corporate financialization. This subsection investigates whether a firm's competitive power plays an important role in this positive relationship. This is because a firm's competitive power likely reduces its financialization, and the firm is inclined to invest in nonfinancial assets because it can gain enough profit from its main business activities due to its high status in the market. The regression is as follows:


(2) $$\begin{array}{l} {\text{Fin}}_{i,t}\;=\;\alpha \;+\;\beta_{1}SC_{i,t}\;+\;\beta_{2}MP_{i,t}\;+\;\beta_{3}MP_{i,t}\;*\;SC_{i,t}\\ \;+\;\beta_{4}Controls_{i,t}\;+\;\beta_{5}Ind\;+\;\beta_{6}Year\;+\;\varepsilon_{i,t}, \end{array}$$

where *MP*_*i, t*_ stands for competitive power which is equal to income minus cost divided by income. The definitions of the other variables are provided in Table 2.

Table 4 reports the results of Equation (2). It provides clear evidence that competitive power negatively impacts a firm's financialization; the estimate of β_2_ is −4.0238 in column (3) and −0.0348 in column (6), consistent with our conjecture. This finding suggests that a firm that has strong market competitive power reduces its investment in financial assets because it can obtain sufficient profitability from its primary business. However, the cross-term for X1 is not robust. The coefficients for X1 (equal to MP multiplied by SC2) is −0.0191 in column (3), the X2 (equal to MP multiplied by CC2) is 0.0139 in column (6). This finding implies that the firm will reduce its investment in financial assets when it obtains enough profit from the main business but cannot reverse the pressure from the supply chain concentration. The firm holds more financial assets when it suffers from a concentrated supply chain; that is, it has a higher supplier or customer concentration, and perhaps the major suppliers or customers erode their profit.

**Table 4:** Supply chain concentration, competitive power, and financialization.

	**(1)**	**(2)**	**(3)**	**(4)**	**(5)**	**(6)**
	**FIN1**	**FIN1**	**FIN1**	**FIN1**	**FIN1**	**FIN1**
SC2	0.0413***	0.0573***	0.0396***			
	(19.4372)	(37.4165)	(18.6204)			
CC2				0.0348***	0.0454***	0.0308***
				(17.0354)	(25.9576)	(14.9112)
MP	−6.4028***	−6.6407***	−4.0238***	−7.9718***	−8.6440***	−4.3866***
	(−7.9530)	(−7.5845)	(−4.7519)	(−9.0790)	(−8.4253)	(−4.7897)
X1	−0.0240	−0.0538***	−0.0191			
	(−0.9709)	(−2.8919)	(−0.7903)			
X2				0.0125	−0.0084	0.0139
				(0.4702)	(−0.3624)	(0.5295)
lev		−6.7301***	−4.8021***		−7.2118***	−4.8413***
		(−22.9563)	(−16.2449)		(−24.4298)	(−16.3522)
ldbl		−0.0779***	0.1536***		−0.1012***	0.1598***
		(−3.9281)	(7.2905)		(−5.0591)	(7.5715)
zzl		−0.0006*	0.0000		−0.0003	0.0004
		(−1.9498)	(0.0561)		(−1.0649)	(1.4170)
zbmjd		0.0010	−0.0110**		−0.0006	−0.0089**
		(0.2547)	(−2.5394)		(−0.1549)	(−2.0640)
roa		−3.6900***	−0.3506		−4.5810***	−0.4614
		(−7.3062)	(−0.5795)		(−8.9953)	(−0.7615)
size		0.4493***	−0.0894**		0.6668***	−0.1029**
		(11.1904)	(−2.2247)		(16.3964)	(−2.5520)
cflow		0.0258	−0.7181***		0.1091	−0.6664***
		(0.1387)	(−3.0186)		(0.5817)	(−2.7965)
top1		−0.0553***	−0.0318***		−0.0544***	−0.0321***
		(−27.0740)	(−9.6635)		(−26.3850)	(−9.7145)
soe		1.8560***	1.8117***		1.7622***	1.7450***
		(15.0904)	(14.1315)		(14.2083)	(13.5890)
age		−0.2040	−2.0e+02***		−0.2108	−2.0e+02***
		(−0.3985)	(−12.4533)		(−0.4115)	(−12.4646)
Firm FE	N	Y	N	N	Y	N
Year FE	Y	Y	Y	Y	Y	Y
Ind FE	Y	N	Y	Y	N	Y
Prov FE	Y	Y	Y	Y	Y	Y
Adj. *R*^2^	0.0909	0.0672	0.1347	0.1091	0.0154	0.1323
*F*	174.1431***	2,988.62***	137.5245***	160.4237***	2,222.18***	128.0005***
*N*	43,564	43,590	43,457	43,457	43,590	43,457

This table shows the relationship between supply chain concentration, competitive power, and firm financialization. The dependent variables and independent variables are described above. We measure competitive power as the market share in the same industry (MP). The cross terms are X1 (equal to SC2 multiplied by MP) and X2 (equal to CC2 multiplied by MP). To avoid multicollinearity, we decentralized SC2, CC2, and MP. All regressions use industry-fixed-effects and year-fixed effects. The industry is classified as the two-digit CSRC industry standard. Continuous variables are winsorized at their 1st and 99th percentiles. Standard errors are corrected for heteroskedasticity and clustering at the firm level (t-statistics are shown in parentheses), *p < 0.1, **p < 0.05, and ***p < 0.01. See Table 2 for the variable definitions.

## Mechanism analysis

To find the mechanism driving the positive relationship between supply chain concentration and corporate financialization, we use a mediation effect analysis (Baron and Kenny, 1986) and explore the causal relationship between them. Specifically, we take three steps: first, we examine whether supply chain concentration (i.e., SC2 and CC2) affects financialization (i.e., FIN1) in model (i); second, we test whether the mediating variable (i.e., OPE or Risk) affects financialization (i.e., FIN1) in model (ii); third, we examine whether the mediating variable (i.e., OPE or Risk) and independent variable (i.e., SC2 and CC2) together affect the dependent variable (i.e., FIN1) in model (iii). Furthermore, to verify that the mediation effect truly exists, we examine the coefficient of the indirect effect using the Sobel test (Sobel, 1982). To clearly show mediation analysis, we estimate the following model:


(i) $$\begin{array}{l} {\text{Fin}}_{i,t}\;=\;\alpha \;+\;c_{1}SC_{i,t}\;+\;\beta_{2}Controls_{i,t}\;+\;\beta_{3}Ind\\ \;+\;\beta_{4}Year\;+\;\varepsilon_{i,t}, \end{array}$$

(ii) $$\begin{array}{l} {\text{SC}}_{i,t}\;=\;\alpha \;+\;aMV_{i,t}\;+\;\beta_{2}Controls_{i,t}\;+\;\beta_{3}Ind\\ \;+\;\beta_{4}Year\;+\;\varepsilon_{i,t}, \end{array}$$

(iii) $$\begin{array}{l} {\text{Fin}}_{i,t}\;=\;\alpha \;+\;c_{2}SC_{i,t}\;+\;bMV_{i,t}\;+\;\beta_{3}Controls_{i,t}\\ \;+\;\beta_{4}Ind\;+\;\beta_{5}Year\;+\;\varepsilon_{i,t}, \end{array}$$

where *Fin*_*i, t*_ is the dependent variable measuring corporate financialization, as mentioned above; *SC*_*i, t*_ is the independent variable measuring the supply chain concentration, including SC2 and CC2; and *MV*_*i, t*_ is the mediating variable measuring *OPE* and *Risk*. The OPE is defined as the main business margin, which equals the main operation income minus the prime operating cost, selling expense, and administration expense divided by the income from the firm's main operations. *Risk* is measured as the standard deviation of the return on assets during the previous and lagged two years based on Boubakri et al. (2013). The coefficients of *a* in model (ii) and *b* in model (iii) are interesting estimates. The indirect effect equals *a* multiplied by *b*(i.e., *ab* is the indirect effect).

### Supply chain concentration impairs the profitability of the main business

In this subsection, we use main business profitability (OPE) as the mediating variable and examine whether the *OPE* has partly or completely mediating effects. This part proves some preconditions for the mediating effect of *OPE*. First, supply chain concentration (i.e., SC2 and CC2) has a significant positive influence on corporate financialization (i.e., the coefficient of *c*_1_ in the total effect model is significant and positive). Second, supply chain concentration has a negative correlation with OPE (i.e., the coefficient of *a* is significant and negative). Finally, the supply chain concentration and OPE together impact corporate financialization (i.e., the coefficients of b and c_2_ are significant, and the coefficient of c_2_ is smaller than that of *c*_1_). In addition, the significance of the mediated effect should be tested using Sobel or bootstrapping (MacKinnon et al., 2012; Hayes and Scharkow, 2013).

As shown in Table 5, the above conditions are not all met, thereby we cannot confirm the mediating role of *OPE* in the relationship between supply chain concentration and corporate financialization. First, supply chain concentration is positively correlated with corporate financialization with *a* coefficient of 0.0393 on SC2 and 0.0314 on CC2 in columns (1) and (3) of Table 6. Second, the supply chain concentration is positively correlated with *OPE* and their coefficient is 0.0175 and 0.0169 as shown in columns (2) and (4) of Table 5, but it is not significant. Furthermore, the SC and *OPE* combined influence financialization, and their coefficients are 0.0395 and−0.0003 as shown in column (3) of Table 5. An indirect effect does not exist. Accordingly, when we use CC2 as the independent variable, we obtain similar results; the indirect effect is ignored. *OPE* does not play a mediating role in the relationship between supply chain concentration and corporate financialization.

**Table 5:** Mediating effect of main business profitability (OPE).

	**(1)**	**(2)**	**(3)**	**(4)**	**(5)**	**(6)**
	**FIN1**	**OPE**	**FIN1**	**FIN1**	**OPE**	**FIN1**
SC2	0.0393***	0.0175	0.0395***			
	(19.0719)	(1.0208)	(19.1738)			
CC2				0.0314***	0.0169	0.0317***
				(15.7138)	(1.0144)	(15.7738)
OPE			−0.0003			−0.0002
			(−0.4452)			(−0.4275)
lev	−4.8151***	−4.6870*	−5.0428***	−4.8601***	−4.7191*	−5.0991***
	(−16.2890)	(−1.9069)	(−17.0266)	(−16.4177)	(−1.9197)	(−17.1908)
ldbl	0.1547***	−0.9618***	0.1423***	0.1601***	−0.9609***	0.1481***
	(7.3391)	(−5.5071)	(6.7587)	(7.5858)	(−5.5032)	(7.0295)
zzl	0.0000	−0.0006	−0.0000	0.0004	−0.0004	0.0004
	(0.0166)	(−0.2343)	(−0.1016)	(1.3753)	(−0.1521)	(1.2696)
zbmjd	−0.0123***	0.0081	−0.0120***	−0.0101**	0.0092	−0.0097**
	(−2.8550)	(0.2276)	(−2.7720)	(−2.3430)	(0.2561)	(−2.2547)
roa	−0.4112	19.3222***	−0.6088	−0.5197	19.2708***	−0.7258
	(−0.6798)	(3.8473)	(−1.0058)	(−0.8581)	(3.8372)	(−1.1976)
size	−0.1691***	0.2622	−0.1406***	−0.1768***	0.2698	−0.1478***
	(−4.5059)	(0.8335)	(−3.7102)	(−4.6861)	(0.8538)	(−3.8756)
cflow	−0.7816***	−0.3464	−0.6690***	−0.7282***	−0.3097	−0.6098**
	(−3.2884)	(−0.1758)	(−2.8171)	(−3.0586)	(−0.1571)	(−2.5634)
top1	−0.0324***	0.0085	−0.0311***	−0.0326***	0.0081	−0.0313***
	(−9.8335)	(0.3124)	(−9.4720)	(−9.8733)	(0.2980)	(−9.5243)
soe	1.8081***	−0.6385	1.8204***	1.7435***	−0.6697	1.7546***
	(14.0997)	(−0.6018)	(14.2400)	(13.5778)	(−0.6313)	(13.7075)
age	−2.1e+02***	−2.3e+02*	−2.1e+02***	−2.1e+02***	−2.3e+02*	−2.1e+02***
	(−12.6188)	(−1.7159)	(−12.5889)	(−12.6353)	(−1.7253)	(−12.6243)
Year FE	Y	Y	Y	Y	Y	Y
Ind FE	Y	Y	Y	Y	Y	Y
Prov FE	Y	Y	Y	Y	Y	Y
Adj. *R*^2^	0.1341	−0.0008	0.1355	0.1317	−0.0008	0.1331
*F*	159.5179***	5.0984***	145.7563***	148.5605***	5.0973***	135.5378***
*N*	43,463	42,962	42,962	43,463	42,962	42,962

This table shows the mediating effect of the main business profitability between supply chain concentration and corporate financialization. Continuous variables are winsorized at their 1st and 99th percentiles. Standard errors are corrected for heteroskedasticity and clustering at the firm level (t-statistics are shown in parentheses), *p < 0.1, **P < 0.05, and ***P < 0.01. See Table 2 for the variable definitions.

**Table 6:** Mediating effect of operating risk (*Risk*).

	**(1)**	**(2)**	**(3)**	**(4)**	**(5)**	**(6)**
	**FIN1**	**Risk**	**FIN1**	**FIN1**	**Risk**	**FIN1**
SC2	0.0393***	0.0047***	0.0205***			
	(19.0719)	(4.9224)	(20.0256)			
CC2				0.0314***	0.0080***	0.0123***
				(15.7138)	(8.6449)	(16.4445)
Risk			0.0315***			0.0293***
			(3.0857)			(2.8672)
lev	−4.8151***	1.7499***	−5.0109***	−4.8601***	1.7358***	−5.0516***
	(−16.2890)	(12.6395)	(−17.1549)	(−16.4177)	(12.5443)	(−17.2667)
ldbl	0.1547***	−0.0293***	0.0761***	0.1601***	−0.0311***	0.0822***
	(7.3391)	(−2.9129)	(3.5962)	(7.5858)	(−3.1001)	(3.8810)
zzl	0.0000	0.0005***	−0.0000	0.0004	0.0006***	0.0004
	(0.0166)	(3.3326)	(−0.0295)	(1.3753)	(3.9150)	(1.4010)
zbmjd	−0.0123***	0.0029	−0.0131***	−0.0101**	0.0032	−0.0108**
	(−2.8550)	(1.4330)	(−3.0682)	(−2.3430)	(1.5923)	(−2.5281)
roa	−0.4112	−10.4886***	−0.1730	−0.5197	−10.5066***	−0.3099
	(−0.6798)	(−36.9580)	(−0.2849)	(−0.8581)	(−37.0439)	(−0.5096)
size	−0.1691***	−0.5054***	−0.1415***	−0.1768***	−0.4907***	−0.1506***
	(−4.5059)	(−28.7184)	(−3.7824)	(−4.6861)	(−27.7816)	(−4.0041)
cflow	−0.7816***	−0.1167	−0.8580***	−0.7282***	−0.0942	−0.8018***
	(−3.2884)	(−1.0498)	(−3.6657)	(−3.0586)	(−0.8474)	(−3.4190)
top1	−0.0324***	−0.0077***	−0.0307***	−0.0326***	−0.0081***	−0.0309***
	(−9.8335)	(−5.0085)	(−9.5034)	(−9.8733)	(−5.2967)	(−9.5489)
soe	1.8081***	−0.1118*	1.6570***	1.7435***	−0.1237**	1.5904***
	(14.0997)	(−1.8671)	(13.1426)	(13.5778)	(−2.0673)	(12.5951)
age	−2.1e+02***	−68.6774***	−2.3e+02***	−2.1e+02***	−70.3228***	−2.4e+02***
	(−12.6188)	(−8.8700)	(−14.3856)	(−12.6353)	(−9.0844)	(−14.4031)
Year FE	Y	Y	Y	Y	Y	Y
Ind FE	Y	Y	Y	Y	Y	Y
Prov FE	Y	Y	Y	Y	Y	Y
Adj. *R*^2^	0.1341	0.1389	0.1379	0.1317	0.1399	0.1353
*F*	159.5179***	388.7625***	139.2847***	148.5605***	393.8127***	128.0788***
*N*	43,463	42,647	42,647	43,463	42,647	42,647

This table shows the mediating effect of operating risk (Risk) between supply chain concentration and corporate financialization. Continuous variables are winsorized at their 1st and 99th percentiles. Standard errors are corrected for heteroskedasticity and clustering at the firm level (t–statistics are shown in parentheses), *P < 0.1, **P < 0.05, and ***P < 0.01. See Table 2 for the variable definitions.

### Supply chain concentration increases firm risk

In this subsection, we use operating risk (*Risk*) as the mediating variable and examine whether *Risk* has partly or completely mediating effects. This part proves some preconditions for the mediating effect of Risk as mentioned above.

As shown in Table 6, all the above conditions are met, thereby confirming the mediating role of *Risk* in the relationship between supply chain concentration and corporate financialization. First, supply chain concentration is positively correlated with corporate financialization with *a* coefficient of 0.0393 on SC2 and 0.0314 on CC2 in columns (1) and (3) of Table 6. Second, supply chain concentration is positively and significantly correlated with operating risk (*Risk*), and their coefficients are 0.0047 and 0.008 in columns (2) and (4), respectively. Furthermore, supply chain concentration and operating risk (*Risk*) together significantly influence financialization, and their coefficients are 0.0205 and 0.0315 in column (3). Accordingly, when we use CC2 as the independent variable, we obtain similar results. Their coefficients are 0.0123 and 0.0293 in column (6). This evidence implies that supply chain concentration, by increasing a firm's operating risk, further affects its financialization level. *Risk* plays a partial mediating role between supply chain concentration and corporate financialization.

In summary, the supply chain concentration increases corporate financialization via one channel, i.e., Risk. The above results show that supply chain concentration increases a firm's operating risk. Therefore, the firm invests in financial assets to spread the risk. Similarly, when focusing on the *OPE* channel, the proportion of the indirect effect of *Risk* is ignored. These findings suggest that the supply chain concentration reduces the firm's main operating profit due to the pressure from upstream firms (suppliers) and downstream firms (customers); thus, the firm switches to holding financial products with short-term and less uncertain value.

## Robustness tests

### Instrumental variable approach

To confirm the main results, we should address endogeneity problems, such as omitted variables, reverse causality, and self-selection bias. Despite the fact that the regressions include a set of firm characteristics control variables, and consider industry and year fixed effects, the coefficient of supply chain concentration might still be biased. A valid instrumental variable can partly alleviate omitted variable issues and reverse causality problems. Thus, we next examine the robustness of our main findings to control for endogeneity using an instrumental variables approach. This approach relies on the notion that our instrumental variables are related to our supply chain concentration measures but uncorrelated with the error terms. That is, a proper instrumental variable must satisfy two conditions (e.g., Roberts and Whited, 2013). First, the relevance condition requires that instruments are correlated with our measures of supply chain concentration after including a set of control variables in our baseline model. Second, the exclusion restriction requires that the instruments are correlated only with measures of supply chain concentration.

First, we select 1-year lagged supply chain concentration (i.e., LSC2 and LCC2) as our instrumental variable based on Dhaliwal et al. (2016). The 1-year lagged supply chain concentration should meet the two conditions mentioned above because it is highly correlated with the current supply chain concentration and likely unrelated to corporate financialization. Second, we perform various tests that suggest that our selected instruments are valid, and Appendix A shows our validity tests. For the weak identification test, the Cragg-Donald Wald F statistic is 5% higher than the maximal IV, which implies that the instruments we use are appropriate. These IVs do not suffer from weak instruments or under-identification issues.

Table 7 presents results that we obtain by regressing each instrumental variable and a set of control variables based on the above-mentioned variables. Consistent with the findings from the baseline regression, the positive relation between supply chain concentration variables (SC2 and CC2) and corporate financialization (FIN1) remains significant at 0.0293 and 0.0072 after taking into account the endogenous issue in columns (1) and (2) with the 1-year lagged supply chain concentration as the IVs (LSC2 and LCC2). The above findings show a positive relationship between supply chain concentration and financialization across all IVs of supply chain concentration. Thus, to the extent that our instruments are valid, the results in Table 7 suggest that higher supply chain concentration causally increases a firm's financialization.

**Table 7:** Instrumental variable regressions.

	**(1)**	**(2)**
	**FIN1**	**FIN1**
SC2	0.0293***	
	(8.20)	
CC2		0.0072***
		(2.63)
lev	−3.0699***	−3.0889***
	(−10.30)	(−10.33)
ldbl	0.1591***	0.1715***
	(6.66)	(7.17)
zzl	0.0018***	0.0020***
	(5.84)	(6.62)
zbmjd	0.0369***	0.0388***
	(8.60)	(9.01)
roa	−0.4799	−0.6112
	(−0.76)	(−0.97)
size	−0.2194***	−0.2756***
	(−5.75)	(−7.26)
cflow	−1.3463***	−1.3418***
	(−5.53)	(−5.50)
top1	−0.0356***	−0.0339***
	(−10.23)	(−9.72)
soe	1.6744***	1.6398***
	(12.83)	(12.53)
age	0.0556	0.0425
	(0.23)	(0.18)
Year FE	Y	Y
Ind FE	Y	Y
Prov FE	Y	Y
Adj. *R*^2^	0.0773	0.0720
*F*	3,206.67***	3,128.66***
*N*	40,142	40,142

This table reports the results from second–stage least squares regressions relating the supply chain concentration to corporate financialization measures and control variables using instrumental variables for lagged one–year supply chain concentration. All other variables are defined in Table 2. Continuous variables are winsorized at their 1st and 99th percentiles. Standard errors are corrected for heteroskedasticity and clustering at the firm level (t–statistics are shown in parentheses), *P < 0.1, **P < 0.05, and ***P < 0.01. *, **, ***denote significance at the 10%, 5%, 1% level, respectively.

### PSM sample analysis

Firms may not randomly have supply chain concentration, as it can be affected by some unobservable factors. To mitigate the resulting bias caused by these differences, we use a propensity score matched (PSM) approach to address this issue (Dehejia and Wahba, 2002). We divide the sample into two groups: (1) a treatment group that contains firms with major suppliers and customers, and (2) a control group that includes firms without large suppliers and customers. The matching variables are firm size (*size*), firm leverage (*lev*), return on total assets (*roa*), the asset turnover ratio (*zzl*), corporate growth ability (*growth*), the share proportion of the largest shareholder (*top1*), and the nature of ownership (*soe*).

First, we use a conditional logit regression model and regress the dummy variable (*dum1*) for whether a firm has a major supplier (major supplier defined as a purchase ratio from the largest supplier of more than 10%, based on previous literature, such as Dhaliwal et al., 2016) and estimate the probability (i.e., the propensity score) that a firm purchases from a large supplier. Panel A of Table 8 presents our logit regression results, which show that the probability of purchasing from a major supplier is significantly positively related to firm leverage (*lev*), liquidity ratio (*ldbl*), asset turnover ratio (*zzl*), firm age (*age*), return on assets (*roa*), firm size (*size*), and the nature of ownership (*soe*) and is negatively correlated with firm size (*size*), return on total assets (*roa*), and the asset turnover ratio (*zzl*).

**Table 8:** Propensity score matched analysis.

**Panel A: Logit regression**									
	**Lev**	**Ldbl**	**Zzl**	**Age**	**roa**	**size**	**Soe**	***N***	*p*−−*R*^2^
dum1	−0.905***	0.013**	−0.001***	−0.151***	−1.630***	−0.117***	−0.363***	43,681	0.015
	(−11.72)	(2.48)	(−2.60)	(−2.90)	(−10.17)	(−12.11)	(−10.59)		
**Panel B: Balancing assumption test of PSM**									
**Variable**	**Unmatched**	**Mean**		**% bias**	**% reduct. Bias**		***t*** **–test**	**V(T)/V(C)**	
	**Matched**	**Treated**	**Control**			***t*** **–value**	***p*** > ***t***		
lev	U	0.39624	0.45161	−25.4		−22.7	0.00	1.00	
	M	0.39624	0.39473	0.7	97.3	0.51	0.61	1.02	
ldbl	U	2.8892	2.3191	20.7		19.59	0.00	1.50*	
	M	2.8892	2.8556	1.2	94.1	0.82	0.41	1.03	
zzl	U	32.183	40.759	−6.3		−5.46	0.00	0.80*	
	M	32.183	31.654	0.4	93.8	0.31	0.76	1.11*	
zbmjd	U	4.7922	5.2234	−4.1		−3.71	0.00	1.06*	
	M	4.7922	4.874	−0.8	81	−0.59	0.56	1.24*	
roa	U	0.03839	0.03989	−1.8		−1.66	0.10	1.17*	
	M	0.03839	0.03864	−0.3	83.6	−0.21	0.83	1.06*	
size	U	21.724	22.068	−25.9		−22.4	0.00	0.76*	
	M	21.724	21.711	1.00	96	0.78	0.43	0.89*	
cflow	U	0.07721	0.08571	−4.8		−4.19	0.00	0.89*	
	M	0.07721	0.07496	1.3	73.5	0.96	0.34	1.10*	
top1	U	21.344	17.601	18.1		16.11	0.00	0.94*	
	M	21.344	21.411	−0.3	98.2	−0.23	0.82	0.86*	
age	U	7.5912	7.5968	−2.6		−2.74	0.01	3.11*	
	M	7.5912	7.5947	−1.6	38	−1.08	0.28	1.73*	
**Panel C: Matching regressions**									
**Variable**	**Sample**		**Treated**		**Controls**	**Difference**		**S.E**.	***T*** **–stat**
Unmatched			6.948		5.551	1.397		0.100	13.97
Near matching (1:2)	ATT		5.618		1.330	0.124		10.75	10.75
Radius matching	ATT		6.957		5.625	1.332		0.105	12.640
Kernel matching	ATT		6.948		5.620	1.328		0.100	13.240
Mahal matching	ATT		6.948		5.574	1.374		0.138	9.930

The table presents the results of PSM analyses. Panel A presents the conditional logit regression, Panel B presents the balancing assumptions test, and Panel C presents a set of matching regression results. Dum1 in Panel A is an indicator variable that equals one if the purchase ratio of the firm's largest supplier is more than 10% and zero otherwise. We adopt near matching, kernel matching, and Mahalanobis matching to estimate the ATTs (i.e., average treatment effects on the treated) S.E. is a standard error, and T–stat is t-statistics. *, **, ***denote significance at the 10%, 5%, 1% level, respectively.

Second, we perform a balancing assumption test based on nearest neighbor matching, following Grilli and Rampichini (2011). Panel B of Table 8 presents the results. After matching, the bias between the treatment group and control group is small, and the grouping bias is below 5%, which satisfies the balancing assumption requirements (Rosenbaum and Rubin, 1985).

Third, we perform a set of matching regressions, such as near matching, radius matching, kernel matching, and Mahalanobis matching, to estimate the ATTs (i.e., average treatment effects on the treated). Panel C of Table 8 reports the results, and the relation between supply chain concentration and corporate financialization is significant and positive. This evidence suggests that our baseline results are confirmed and that our findings are robust.

### Alternative measurements

To perform further robustness checks of our baseline results, we first use the ratio of financial income to total income as an alternative corporate financialization measure. We report the corresponding results in columns (1) and (2) of Table 9. With the alternative measure, the SC-financialization relation remains significant and positive with a coefficient of 0.2254 on SC2 and 0.3627 on CC2. We then use the purchase/buy ratio of the largest supplier or customer as the independent variable (i.e., SC1 and CC1) and then regress SC1/CC1 on corporate financialization. The results are as reported in columns (1) and (2) of Table 9. With the alternative measures, the relation remains significant and positive, with the coefficient of 0.0487 on SC1 and 0.0348 on CC1 in columns (3) and (4). Last, we use the sum of SC2 and CC2 as an alternative supply chain concentration (SLC), then regress the FIN1 on SLC, the coefficient is 0.0051. The above results show clear evidence supporting our hypothesis that supply chain concentration positively affects firm financialization.

**Table 9:** Alternative independent variables.

	**(1)**	**(2)**	**(3)**	**(4)**	**(5)**
	**FIN2**	**FIN2**	**FIN1**	**FIN1**	**FIN1**
SC2	0.2254**				
	(2.5333)				
CC2		1.3627***			
		(3.2140)			
SC1			0.0487***		
			(12.4741)		
CC1				0.0348***	
				(10.2823)	
SLC					0.0551***
					(21.4392)
lev	−17.1858	−21.0984	−4.8090***	−4.8540***	−4.8551***
	(−0.2708)	(−0.3325)	(−16.2296)	(−16.3692)	(−16.4416)
ldbl	4.6441	5.2308	0.1700***	0.1684***	0.1468***
	(1.0280)	(1.1580)	(8.0542)	(7.9738)	(6.9690)
zzl	−0.0343	−0.0454	0.0001	0.0003	0.0003
	(−0.5468)	(−0.7224)	(0.4417)	(0.9131)	(0.8760)
zbmjd	0.1385	0.1699	−0.0114***	−0.0103**	−0.0114***
	(0.1664)	(0.2043)	(−2.6420)	(−2.3837)	(−2.6408)
roa	−65.7113	−68.8708	−0.4246	−0.5044	−0.4657
	(−0.4766)	(−0.4996)	(−0.7003)	(−0.8314)	(−0.7708)
size	19.7916**	15.1635*	−0.2311***	−0.2466***	−0.1091***
	(2.4071)	(1.8376)	(−6.1839)	(−6.6035)	(−2.8796)
cflow	7.7650	4.1033	−0.8491***	−0.8327***	−0.7002***
	(0.1592)	(0.0841)	(−3.5643)	(−3.4934)	(−2.9485)
top1	0.2417	0.4045	−0.0301***	−0.0304***	−0.0343***
	(0.3522)	(0.5889)	(−9.1358)	(−9.2277)	(−10.4073)
soe	10.1069	10.6514	1.8261***	1.7698***	1.7750***
	(0.3955)	(0.4170)	(14.1990)	(13.7614)	(13.8580)
age	−1.0e+03	−6.6e+02	−2.1e+02***	−2.0e+02***	−2.1e+02***
	(−0.2850)	(−0.1807)	(−12.5959)	(−12.4011)	(−13.0255)
Year FE	Y	Y	Y	Y	Y
Ind FE	Y	Y	Y	Y	Y
Prov FE	Y	Y	Y	Y	Y
Adj. *R*^2^	0.0010	0.0014	0.1299	0.1289	0.1360
*F*	0.7492	1.6627*	139.9943***	135.3158***	168.5141***
*N*	26445	26445	43463	43463	43463

This table reports the results of alternative measurements for the independent variables. In particular, we use SC1 (the purchase ratio of the largest supplier) and CC1 (the sales ratio of the largest customer) instead of SC2 and CC2. All regressions use industry–fixed effects and year–fixed effects, the industry category based on a firm's 3–digit CSRC industry classification. All other variables are defined in Table 2. Continuous variables are winsorized at their 1st and 99th percentiles. Standard errors are corrected for heteroskedasticity and clustering at the firm level (t–statistics are shown in parentheses), *P < 0.1, **P < 0.05, and ***P < 0.01.

One may argue that high-financialization firms choose to concentrate their large suppliers and/or customers, while low-financialization firms choose to have dispersed suppliers and/or customers to gain more credit, so low-financialization firms' financialization could negatively affect the supply chain concentration (i.e., reverse causality). To ascertain the possible asymmetric impact of high financialization on the supply chain concentration and financialization relation, we divide the sample into high financialization and low financialization based on the median of the ratio of financial assets to total assets (i.e., *FIN1*). The subsample results reported in Table 10 show that the positive relationship between supply chain concentration and corporate financialization is present in low financialization subsamples. This regression favorably supports that our findings presented are not due to reverse causality.

**Table 10:** Grouped regressions.

	**(1) Fin**	**(2) Fin**	**(3) Fin**	**(4) Fin**
	**Low–Fin**	**High–Fin**	**Low–Fin**	**High–Fin**
SC2	0.0391***	−0.0649***		
	(137.876)	(−20.826)		
CC2			0.0297***	−0.0574***
			(102.508)	(−18.780)
lev	−0.1044***	−3.2246***	−0.5782***	−3.0127***
	(−2.872)	(−5.738)	(−14.370)	(−5.344)
ldbl	−0.0061**	0.4409***	−0.0257***	0.4259***
	(−2.265)	(10.973)	(−8.549)	(10.585)
zzl	−0.0003***	0.0030***	0.0000	0.0015**
	(−8.934)	(4.851)	(0.880)	(2.383)
zbmjd	0.0022***	0.0442***	0.0030***	0.0373***
	(3.995)	(5.783)	(4.980)	(4.859)
roa	−0.1721**	−0.6464	−0.4165***	−0.2123
	(−2.224)	(−0.551)	(−4.844)	(−0.181)
Size	0.0315***	−0.3380***	0.0912***	−0.3371***
	(7.668)	(−4.833)	(19.736)	(−4.799)
cflow	−0.1633***	−1.6574***	−0.0402	−1.5929***
	(−5.546)	(−3.439)	(−1.229)	(−3.297)
top1	0.0012***	−0.0738***	0.0009***	−0.0772***
	(4.251)	(−14.794)	(2.962)	(−15.503)
soe	0.0978***	1.5261***	−0.0622***	1.8031***
	(6.497)	(5.904)	(−3.730)	(6.970)
Age	−0.0088	0.1301	−0.0169	−0.0178
	(−0.286)	(0.329)	(−0.492)	(−0.045)
Year FE	Y	Y	Y	Y
Ind FE	Y	Y	Y	Y
Prov FE	Y	Y	Y	Y
Adj. *R*^2^	0.438	0.063	0.305	0.058
*F*	1,804.087***	110.858***	1,016.683***	103.179***
*N*	25,485	18,111	25,485	18,111

This table reports the group regression. We divide the sample into high– and low–financialization firms based on the median ratio of financial assets to total assets (i.e., Fin1). All regressions use industry–fixed effects and year–fixed effects. The industry is classified by the 3–digit CSRC industry standard. Continuous variables are winsorized at their 1st and 99th percentiles. Standard errors are corrected for heteroskedasticity and clustering at the firm level (t–statistics are shown in parentheses), *p < 0.1, **p < 0.05, and ***p < 0.01. See Table 2 for the variable definitions. *, **, ***denote significance at the 10%, 5%, 1% level, respectively.

## Further tests

To investigate whether supply chain concentration affects a firm's R&D investment and M&A activity, we run a set of regressions based on R&D and M&As. We measure R&D investment by R&D expenditure. We define M&A as a dummy variable that equals one if the firm engages in M&A activity and zero otherwise. We run a multiple regression in which R&D is the dependent variable and run a linear probit model in which M&A is the explained variable due to its use as an indicator variable. Table 11 reports the results. The results show that supply chain concentration reduces R&D investment and increases M&A activity. For example, the coefficient on CC2 is −0.0069 in column (2) when R&D is used as the explained variable, and the coefficient on SC2 is 0.0014 in column (3) when M&A is used as the dependent variable. The results indicate that when a company's purchases or sales are largely concentrated in one or several large suppliers or customers, respectively, the company reduces its investment in R&D and increases M&A activity.

**Table 11:** Supply chain concentration, R&D investment, and M&A activity.

	**(1)**	**(2)**	**(3)**	**(4)**
	**RDI**	**RDI**	**MA**	**MA**
SC2	0.0005		0.0014***	
	(0.2296)		(3.7027)	
CC2		−0.0069***		0.0012***
		(−3.0036)		(3.0920)
lev	−0.7252**	−0.7001*	−0.2133***	−0.2146***
	(−2.0044)	(−1.9349)	(−3.6606)	(−3.6823)
ldbl	−0.0285	−0.0244	−0.0324***	−0.0322***
	(−1.2958)	(−1.1068)	(−7.5477)	(−7.5052)
zzl	−0.0002	−0.0002	−0.0001	−0.0001
	(−0.3252)	(−0.3878)	(−1.4601)	(−1.1833)
zbmjd	0.0164***	0.0165***	−0.0001	0.0000
	(2.6485)	(2.6719)	(−0.1114)	(0.0048)
roa	2.6501***	2.6568***	−1.3467***	−1.3501***
	(3.3574)	(3.3671)	(−10.4239)	(−10.4499)
Size	−0.1923***	−0.2218***	0.0758***	0.0758***
	(−3.9072)	(−4.4912)	(9.9686)	(9.9233)
cflow	0.7380**	0.7007**	−0.0763*	−0.0733
	(2.4106)	(2.2878)	(−1.6969)	(−1.6289)
top1	0.0002	0.0005	−0.0021***	−0.0022***
	(0.0627)	(0.1649)	(−3.6687)	(−3.6963)
soe	0.3393***	0.3393***	−0.2885***	−0.2904***
	(2.7119)	(2.7156)	(−12.7809)	(−12.8632)
Age	49.7336***	51.1608***	11.1855***	11.2832***
	(2.6316)	(2.7071)	(3.1998)	(3.2282)
Year FE	Y	Y	Y	Y
Ind FE	Y	Y	Y	Y
Prov FE	Y	Y	Y	Y
Adj. *R*^2^	0.0307	0.0311	0.2236	0.2235
*F* (or LR chi^2^)	46.8928***	37.7111***	9,483.33***	9,479.19***
*N*	20,369	20,369	34,498	34,498

This table reports whether supply chain concentration affects corporate R&D investment and M&A activity. The independent variable is supply chain concentration, which is measured by SC2 and CC2. One of the dependent variables is R&D investment, which equals research and development expenditure, and the other dependent variable is M&A activity, which equals one if the firm engages in M&A activity and zero otherwise. Other variable definitions are provided in Appendix A. All regressions use industry– and year–fixed effects. The industry is classified by the 3–digit CSRC industry standard. Continuous variables are winsorized at their 1st and 99th percentiles. Standard errors are corrected for heteroskedasticity and clustering at the firm level (t-statistics are shown in parentheses), *P < 0.1, **P < 0.05, and ***P < 0.01.

## Conclusion

Due to the recession of the real economy, an increasing number of nonfinancial firms put their money into the financial and real estate industries. In addition, a firm's operating activity is seriously impacted by supplier and customer relationships. However, how a firm's supplier and customer relationships affect its financialization is unclear. To fill this gap, we study the relation between a firm's supply chain concentration and its financialization using a sample of Chinese listed firms from 2007 to 2021. We further test how a firm's market competitive power affects the correlation between supply chain concentration and corporate financialization. Moreover, we explore the mechanism by which supply chain concentration impacts corporate financialization. To confirm the main results, we use a PSM analysis, IVs regression, and alternative variable measurements to re-examine the baseline model.

We find that there is a significant positive relationship between a firm's supply chain concentration and its financialization, implying that a firm that over-relies on a large supplier or customer increases its financialization; that is, the firm will hold more financial assets to confront pressure from upstream and downstream firms. Furthermore, we find that a firm with strong market competitive power reduces its investment in financial assets because it can obtain sufficient profitability from its primary business. This finding suggests that the market power of firms will alleviate supply chain concentration and reduce financialization, but firms cannot resist pressure from a concentrated supply chain. We further explore the underlying mechanism of the impact of supply chain concentration on corporate financialization. We find that supply chain concentration increases a firm's operating risk; thus, the firm turns to invest in financial assets due to the short payback period and low uncertainty of these assets. Finally, we test how supply chain concentration impacts a firm's R&D investment and M&A activity, further verifying that a firm's dependence on a large supplier or customer may have an adverse impact on the firm. A firm should cope with the relationship between major suppliers and customers along the supply chain.

## Data availability statement

The original contributions presented in the study are included in the article/supplementary material, further inquiries can be directed to the corresponding author.

## Author contributions

MZ is responsible for theoretical literature review, article writing, and adjusting. XZ is responsible for the construction and later revision of this paper. Both authors contributed to the article and approved the submitted version.

## Funding

This research was funded by the General Project of the National Natural Science Foundation of China (72171162), Taiyuan University of Science and Technology Scientific Research Initial Funding (TYSUT SRIF: 20222004), the Key Research Bases for Humanity and Social Science of Shanxi Province (20210132), and the Teaching Reform and Innovation Project of Taiyuan University of Science and Technology (XJ2021104).

## Conflict of interest

The authors declare that the research was conducted in the absence of any commercial or financial relationships that could be construed as a potential conflict of interest.

## Publisher's note

All claims expressed in this article are solely those of the authors and do not necessarily represent those of their affiliated organizations, or those of the publisher, the editors and the reviewers. Any product that may be evaluated in this article, or claim that may be made by its manufacturer, is not guaranteed or endorsed by the publisher.

## Appendix A

**Table A1:** Validity test of the instrumental variables.

**Item**	**L1.SC2**	**L1.CC2**
Under-identification test: Anderson canon. corr. LM statistic (*P*-value)	2,212.504 (0.00)	2,250.276 (0.00)
Weak identification test: Cragg-Donald Wald *F*-statistic	2,800.421	2,407.452
Stock-Yogo weak ID test critical values: 10% maximal IV	16.38	16.38
Overidentification test: Sargan statistic	0.1502	0.1716
